# Laryngeal recurrence of extranodal NK/T cell lymphoma

**DOI:** 10.1002/jha2.233

**Published:** 2021-05-29

**Authors:** Takumi Kumai, Kan Kishibe, Tatsuya Hayashi, Yasuaki Harabuchi

**Affiliations:** ^1^ Department of Innovative Head and Neck Cancer Research and Treatment (IHNCRT), Asahikawa Medical University Asahikawa Japan; ^2^ Department of Otolaryngology, Head and Neck Surgery Asahikawa Medical University Asahikawa Japan

A 72‐year‐old man presented with a 1‐year history of painless progressive nasal obstruction. Nasal fiberscope evaluation revealed a necrotic tumor of the nasal septum, and histopathological evaluation of a biopsy specimen showed significant polymorphous lymphocytic infiltration of the nasal mucosa along with necrosis. The patient was diagnosed with extranodal NK/T cell lymphoma (ENKTL) based on immunohistochemical evaluation that revealed lymphocytes showing positivity for CD3, Granzyme B, CD56, and EB virus‐encoded small RNA (EBER). Fiberscopic examination, fluorodeoxyglucose‐positron emission tomography computed tomography (FDG‐PET/CT), and bone marrow biopsy did not reveal distant metastasis including laryngeal lesions. He was treated with concurrent chemoradiotherapy using an MPVIC‐P regimen (methotrexate, peplomycin, etoposide, ifosfamide, carboplatin, and prednisolone) [1] that led to remission of the nasal lesion.

The patient presented with sore throat, dysphonia, and dyspnea, 3 years after his initial treatment. Flexible endoscopy revealed right vocal cord swelling with subglottic stenosis (Figure 1A). We performed tracheostomy, and histopathological evaluation of a biopsy specimen of the subglottis showed an atypical lymphocytic infiltrate comprising cells that were positive for CD3, Granzyme B, CD56, and EBER, which indicated recurrent ENKTL (Figure 1B). Bone marrow biopsy and FDG‐PET/CT did not reveal metastasis or nasal recurrence; however, we observed high tracer uptake in the larynx (Figure 1C). He was re‐treated with concurrent chemoradiotherapy using the MPVIC‐P regimen and showed no recurrence during 10‐month follow‐up (Figure 1D).

**FIGURE 1:**
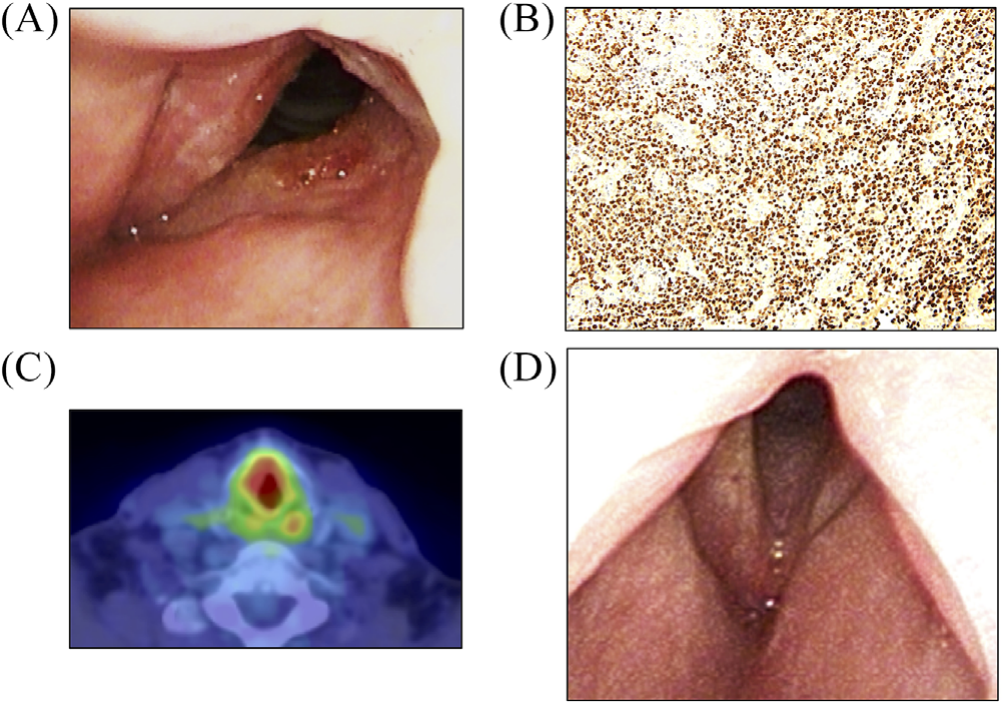
(A) Flexible endoscopy showing a swollen right vocal cord with subglottic stenosis. (B) Histopathological findings of a biopsy specimen of the larynx. Infiltration of atypical lymphocytes is visualized with cells showing positivity for EBER. (C) PET/CT scan showing laryngeal FDG uptake. (D) Image showing subsidence of right vocal fold swelling after treatment

ENKTL is an Epstein Barr virus‐induced malignancy, which commonly affects the upper respiratory tract, particularly the nasal cavity, and laryngeal ENKTL is rare (incidence of laryngeal ENKTL is 1.7%) [2, 3]. Notably, primary laryngeal ENKTL has been reported in <40 patients in the literature, and no studies have described laryngeal involvement in recurrent nasal ENKTL. However, laryngeal involvement in recurrent ENKTL, followed by dyspnea could be fatal; therefore, clinicians should closely monitor throat‐related symptoms such as dysphonia and dyspnea during follow‐up of ENKTL.

## CONFLICT OF INTEREST

The authors declare that there is no conflict of interest that could be perceived as prejudicing the impartiality of the research reported.
